# Kinetic GFR Outperforms CKD-EPI for Slow Graft Function Prediction in the Immediate Postoperative Period Following Kidney Transplantation

**DOI:** 10.3390/jcm9124003

**Published:** 2020-12-10

**Authors:** Jonathan Dash, Thomas Verissimo, Anna Faivre, Lena Berchtold, Thierry Berney, Jérôme Pugin, Sophie de Seigneux, David Legouis

**Affiliations:** 1 Division of Internal Medicine, Department of Medicine, University Hospitals of Geneva, 1205 Geneva, Switzerland; Jonathan.dash@hcuge.ch; 2 Laboratory of Nephrology, Department of Medicine, University Hospitals of Geneva, 1205 Geneva, Switzerland; thomas.verissimo@unige.ch (T.V.); anna.faivre@unige.ch (A.F.); sophie.deseigneux@hcuge.ch (S.d.S.); 3 Department of Cell Physiology, Faculty of Medicine, University of Geneva, 1205 Geneva, Switzerland; 4 Division of Nephrology, Department of Medicine, University Hospitals of Geneva, 1205 Geneva, Switzerland; lena.berchtold@hcuge.ch; 5 Division of Transplantation, Department of Surgery, University Hospitals of Geneva, 1205 Geneva, Switzerland; thierry.berney@hcuge.ch; 6 Division of Intensive Care, Department of Acute Medicine, University Hospital of Geneva, 1205 Geneva, Switzerland; jerome.pugin@hcuge.ch

**Keywords:** transplantation, GFR, CKD-EPI

## Abstract

Background: Rapid identification of patients at high risk for slow graft function (SGF) is of major importance in the immediate period following renal graft transplantation, both for early therapeutic decisions and long-term prognosis. Due to the high variability of serum creatinine levels after surgery, glomerular filtration rate (GFR) estimation is challenging. In this situation, kinetic estimated GFR (KeGFR) equations are interesting tools but have never been assessed for the identification of SGF patients. Methods: We conducted a single-center retrospective cohort study, including all consecutive kidney allograft recipients in the University Hospitals of Geneva from 2008 to 2016. GFR was estimated using both CKD-EPI and KeGFR formulae. Their accuracies for SGF prediction were compared. Patients were followed up for one year after transplantation. Results: A total of 326 kidney recipients were analyzed. SGF occurred in 76 (23%) patients. KeGFR estimation stabilized from the day following kidney transplantation, more rapidly than CKD-EPI. Discrimination ability for SGF prediction was better for KeGFR than CKD-EPI (AUC 0.82 and 0.66, *p* < 0.001, respectively). Conclusion: KeGFR computed from the first day after renal transplantation was able to predict SGF with good discrimination, outperforming CKD-EPI estimation. SGF patients had lower renal graft function overall at the one-year follow up.

## 1. Introduction

Kidney transplantation is the current best treatment for end-stage renal disease [1]. Delayed graft function, defined as the need for dialysis within the first week post-transplantation [2], remains a major issue in the immediate postoperative period, both associated with lower graft survival [3] and mortality [4]. Incidence of delayed graft function is closely related to donor type: approximately fifty percent in donation after cardiac death [5] and approximately 25% in brainstem death donation [4,6], it falls to approximately three percent in grafts from living donors [7,8]. This stringent definition, however, leads to overlooking many patients with a reduced initial renal function who do not meet criteria for dialysis. Slow graft function (SGF) defines patients with a lower creatinine reduction rate in the first few days following kidney transplantation, without the need for renal replacement therapy, describing an intermediate phenotype between immediate and delayed graft function [9]. This presentation is associated with the worst short and long term renal graft function, as well as decrease in graft survival, compared to graft with immediate graft function [10,11]. Rapid prediction of SGF is important for the transplantation team as it might influence therapeutic decisions such as reducing exposure to calcineurin inhibitors, avoiding nephrotoxic medications or optimizing volume status, all strategies that have been suggested to be useful in the context of kidney transplantation [12]. Prediction of SGF may help to better identify patients with long-term grafts and patients’ outcomes [11]. However, predicting SGF rapidly after transplantation remains challenging. Anuria after transplantation is a specific variable, but its sensitivity is low [13]. Change in serum creatinine in the first 24 h after transplantation is not reliable to predict delayed graft function [14]. In addition, formulae to estimate the glomerular filtration rate (eGFR) such as CKD-EPI are not validated when serum creatinine levels vary rapidly [15]. Based on mass-balanced equations and using serum creatinine measured at two different time points, kinetic eGFR (KeGFR) equations are emerging as interesting tools to evaluate renal function when glomerular filtration rate changes abruptly, while serum creatinine needs time to reach a new steady state [16]. Kinetic eGFR has been shown to more accurately predict acute kidney injury and need for renal replacement therapy than steady-state equation [17] and to outperform other biomarkers for renal recovery and major adverse kidney events prediction [18].

In the post-transplantation setting, KeGFR could be of great interest as the serum creatinine levels are not stable [19]. One study has shown that KeGFR performed reasonably well to predict early delayed graft function [20]. Whether the KeGFR formula could predict SGF is unknown. Here we compared the estimation of GFR by CKD-EPI and KeGFR formulae computed in the first five days following kidney transplantation to predict SGF in 326 patients who underwent kidney transplantation in our center during an 8 year period.

## 2. Materials and Methods 

### 2.1. Study Design

We conducted a single-center retrospective cohort study at Geneva University Hospitals, Switzerland. Patients were included from January 2003 to December 2016. All kidney allograft recipients older than 18 years old were enrolled. Patients were secondarily excluded if the recorded serum creatinine measurements were not sufficient to calculate eGFR and define the status “slow graft function”. This study was approved by the ethical committee for human studies of Geneva, Switzerland (CCER 201-00320, Commission Cantonale d’Ethique de la Recherche), and performed according to the Declaration of Helsinki principles.

### 2.2. Definitions

Slow graft function (SGF) was defined as a creatinine reduction rate >20% at postoperative day (POD) 3 with a POD3 serum creatinine level greater than 132 µmol/L [11]. The creatinine reduction rate was calculated according to this equation:

CRR = 100 × (POD1SCr − POD3SCr)/(POD1SCr)

### 2.3. Data Sources

This study was completed using the intensive care unit database from Geneva University Hospitals, updated with all serum creatinine values measured during the 12 months following discharge. All data were anonymized and analyzed in a blinded manner. 

### 2.4. eGFR Calculations

Serum creatinine concentrations were measured using the Jaffé method with isotope dilution mass spectrometry (ID/MS) standardization. CKD-EPI was calculated as described. KeGFR was calculated using Chen’s formula [16]:

KeGFR = (1 − (24 × (CreatTb − CreatTa)/∆time × Max∆PCr)) × SSPCr × CrCl/(0.5 × (CreatTb + CreatTa))

where CreatTa and Creat Tb are the serum creatinine levels at time a and b, respectively, ∆time is the time difference between the two serum creatinine samples, SSPCr is the first serum creatinine level recorded after transplantation, and CrCl is the associated eGFR calculated using the CKD-EPI equation. The Max∆PCr per day was the highest expected increase in serum creatinine in a day and was set at 133 µmol/L/day as proposed by Chen [16]. KeGFR was calculated if and only if ∆time was less than 36 hrs and was expressed in mL/min/1.73 m^2^. Renal function during the follow up was estimated using CKD-EPI equation.

### 2.5. Statistical Analysis 

Continuous variables for individuals were expressed as the mean ± standard error (SE) or as the median and the 25th–75th percentile for non-normally distributed variables. Comparisons between groups were performed using the Wilcoxon rank-sum test.

Categorical variables were expressed as the absolute and relative (%) frequency and compared using Fisher’s exact test.

eGFR and c-statistic were expressed as the mean ±95% confidence interval estimated by bootstrapping. ROC curves were compared by the log-rank test.

Individual trajectories of eGFR was fitted using a linear model.

A *p*-value below 0.05 was considered significant, and all *p*-values were two tailed. *p*-values were adjusted for multiple tests using Holm methods when necessary. Statistical analyses were performed using R software.

## 3. Results

### 3.1. Baseline Characteristics and Outcomes

**Figure 1:**
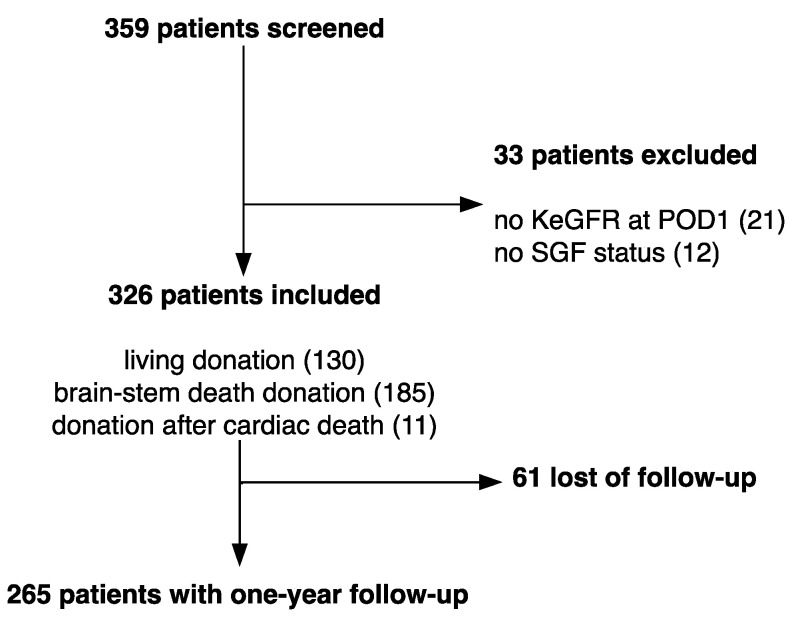
Flowchart of this study.

From January 2003 to December 2016, 359 patients older than 18 years old who underwent a kidney allograft in the Geneva University Hospitals were screened. We excluded 21 (6%) patients as KeGFR estimations could not be performed at POD 1. Finally, we excluded 12 (4%) patients as the SGF status could not be assessed due to missing creatinine values. We finally analyzed 326 patients (Figure 1). In this population, the median age was 53 (ranging from 43 to 63) years old with 199 men and 127 women. Patients’ characteristics are shown in Table 1. Living donor kidney transplantation was performed in 130 (40%) patients. Donors included both brain-stem death donor (*n* = 185, 57%) and donation after cardiac death (*n* = 11, 3%). Median eGFR at 12 months using CKD-EPI equation was 54 (44–67) mL/min/1.73 m^2^. SGF occurred in 76 (23%) patients. Comparatively to the kidney recipients who did not experience SGF, patients displaying SGF were older (58 versus 52 years, *p* = 0.009), received an allograft less frequently from a living donor (16% versus 48%, *p* < 0.001), which donors were also generally older (57 versus 52 years old, *p* = 0.017) and needed more frequently dialysis (0.8% versus 6.6%, *p* = 0.003). In patients with SGF, 3 month, 6 month and one-year estimated GFRs after transplantation were lower (42 (32–63) mL/min/1.73 m^2^ versus 56 (48–68) mL/min/1.73 m^2^, 46 (31–60) mL/min/1.73 m^2^ versus 54 (45–69) mL/min/1.73 m^2^, 47 (38–60) mL/min/1.73 m^2^ versus 57 (45–69) mL/min/1.73 m^2^, respectively, *p* < 0.005). 

### 3.2. KeGFR and CKD-EPI in the Immediate Post-Transplantation Period

**Figure 2:**
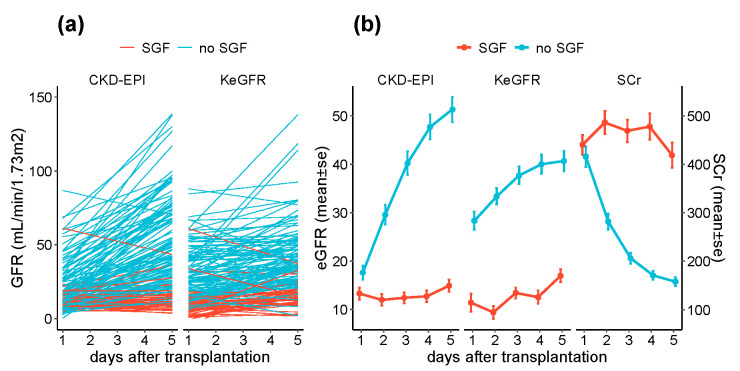
KeGFR and CKD-EPI after transplantation. (**a**) Individual trajectory of GFR estimation using CKD-EPI and KeGFR formulae, stratified on the slow graft function status, and (**b**) pooled CKD-EPI and KeGFR estimations and serum creatinine levels over the next five days after surgery, depending on slow graft function status. Data are shown as the mean ± SE.

Over the five first days after transplantation and independently of the SGF status, KeGFR slightly increased over time (+2.7 mL/min/1.73 m^2^/day, *p* < 0.001). CKD-EPI evolved differently. Although it did not increase in SGF patients (+0.1 mL/min/1.73 m^2^/day, *p* = 0.7), patients without SGF displayed a rapid increase in eGFR (+9.2 mL/min/1.73 m^2^/day, *p* < 0.001) following the serum creatinine levels (−61 µmol/L/day, *p* < 0.001 for no SGF patients) (Figure 2a,b). 

### 3.3. POD1 KeGFR and CKD-EPI According to SGF Status

**Figure 3:**
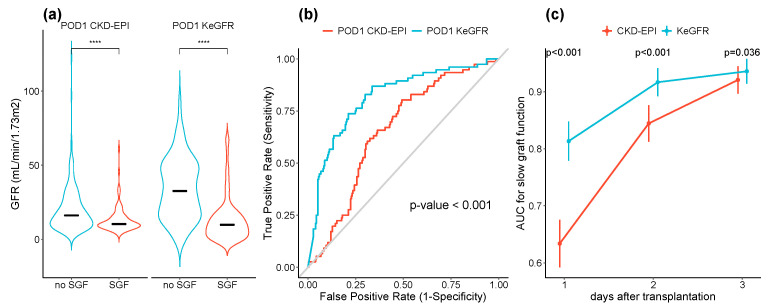
Slow graft function and eGFR. (**a**) Violin plots showing the distribution and the median (black line) of postoperative day 1 (POD1) KeGFR and CKD-EPI eGFRs according to slow graft function groups, (**b**) Receiver Operating Characteristic (ROC) curve for slow graft function prediction using KeGFR (blue) or CKD-EPI (red) calculated at postoperative day 1. Area under cover was compared using the Delong method and (**c**) the area under cover of the ROC curve for slow graft function using KeGFR (blue) and CKD-EPI (red) calculated in the first five days after transplantation. Data are shown as the mean ± 95% confidence interval. **** *p* value < 0.0001.

Median POD1 KeGFR and CKD-EPI were lower in patients experiencing slow graft function (10 (4–17) versus 33 (17–49) mL/min/1.73 m^2^, *p* < 0.0001 and 10 (8–15) versus 16 (10–26) mL/min/1.73 m^2^, *p* < 0.001, respectively) (Figure 3a) but the difference was significantly higher for the KeGFR (*p* < 0.001). At POD 1, the area under the ROC curve for SGF prediction was better for KeGFR compared to CKD-EPI ((AUC 0.82, 95% CI [0.76;0.87]) and (AUC 0.66, 95% CI [0.60;0.72], *p* < 0.001) for GFR estimated using KeGFR or CKD-EPI respectively). Using POD1 KeGFR, the optimal cut-off point giving by the ROC01 index was 15 mL/min/1.73 m^2^ and predicted SGF with a sensitivity of 0.79, a specificity of 0.74, and a positive predictive value of 0.91. The optimal cut-off point for CKD-EPI was 12 mL/min/1.73 m^2^ and predicted SGF with a sensitivity of 0.64, a specificity of 0.66 and a positive predictive value of 0.86 (Figure 3b). 

We then considered the 262 patients who had KeGFR and GFR recorded each in the three first postoperative days following transplantation. During the whole period, KeGFR better discriminated the SGF and non-SGF groups than CKD-EPI eGFR. (Figure 3c).

### 3.4. POD1 eGFR, SGF and Renal Function over Follow Up

**Figure 4:**
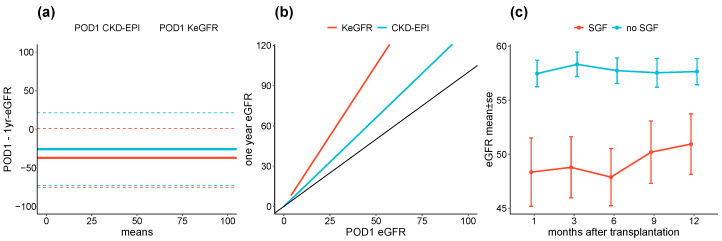
KeGFR and CKD-EPI at POD1 and one-year eGFR. (**a**) Bland–Altman plot comparing eGFR one year after transplantation calculated using CKD-EPI and postoperative day 1 (POD1) GFR estimated either by KeGFR (blue) or CKD-EPI (red). Plain lines showed limits of agreement and dot lines showed biases, and (**b**) GFR estimated using CKD-EPI one year after renal transplantation according to POD1 GFR estimated either by KeGFR (blue) or CKD-EPI (red). (**c**) GFR estimated using CKD-EPI in the first year following renal transplantation, according to slow graft function status.

The one-year eGFR follow up was available for 265 patients. Bland–Altmann analyses comparing both postoperative day 1 estimated GFR and one-year CKD-EPI eGFR reported mean biases of −26 mL/min/1.73 m^2^ LOF [−73;21] for KeGFR and −37 mL/min/1.73 m^2^ LOF [−75;1] for CKD-EPI (Figure 4a). The linear slope between one-year eGFR and POD1 eGFR were 1.3 95%CI [1.2;1.4] and 2.1 95%CI [1.9;2.3] for KeGFR and CKD-EPI equations respectively (Figure 4b). During the one-year follow up, CKD-EPI eGFR did not change over time irrespective of the SGF status (0 mL/min/1.73 m^2^/each 3 months, *p* = 0.1). By contrast, patients who had initially experienced SGF had lower renal function during the entire follow-up period (−10 mL/min/1.73 m^2^, *p* < 0.001) (Figure 4c).). Using multivariable linear regression, SGF status associated significantly with one-year eGFR but not recipients age and sex or type of kidney donation.

### 3.5. Sensitivity Analyses

**Figure 5:**
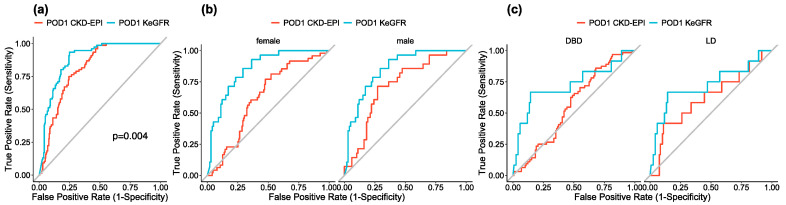
Sensitivity analyses. (**a**) Receiver Operating Characteristic (ROC) curve for slow graft function prediction using KeGFR (blue) or CKD-EPI (red) calculated at POD 1 and defined either by a serum creatinine level greater than 264 µmol/L by postoperative day 5. (**b**) Receiver Operating Characteristic (ROC) curve for slow graft function prediction using KeGFR (blue) or CKD-EPI (red) calculated at postoperative day 1 for female (left panel) and male (right panel). (**c**) Receiver Operating Characteristic (ROC) curve for slow graft function prediction using KeGFR (blue) or CKD-EPI (red) calculated at postoperative day 1 for DBD (left panel) and LD (right panel).

When defining SGF as a serum creatinine level greater than 264 µmol/L by POD 5, we found discrimination ability for predicting this entity to be better with KeGFR than CKD-EPI (AUC = 0.88 95% CI [0.85;0.92] and 0.81 95% CI [0.76;0.86], respectively, *p* = 0.004) (Figure 5a). At POD 1, the area under the ROC curve for SGF prediction was better for KeGFR compared to CKD-EPI in both male and female patients (AUC 0.80 versus 0.63 for females, *p* < 0.001 and AUC 0.85 versus 0.72, *p* < 0.001 for males) (Figure 5b) as well as in donation after brain death (DBD) and living donation (LD), (AUC 0.78 versus 0.56, *p* < 0.001 for DBD and AUC 0.72 versus 0.62 for LD, *p* = 0.04, with KeGFR and CKD-EPI respectively) (Figure 5c).

## 4. Discussion

In this retrospective analysis, we compared the estimation of GFR using CKD-EPI and KeGFR formulae in the five days following renal transplantation to predict SGF in kidney allograft patients. We confirmed the high incidence of SGF, occurring in one over five patients, and its association to a mid-term worse renal prognosis. KeGFR estimation was stable from the day following surgery and did not change in a clinically significant manner in the next five days. On the other hand, CKD-EPI increased all over the five first days. KeGFR was more discriminative to predict SGF than CKD-EPI from POD1 to POD3.

In this work, we showed that SGF is a frequent condition occurring in 23% of patients, more in grafts originating from donation after death than in kidneys from living donors (33% versus 9%, respectively, *p* < 0.001). This is in line with the incidence of SGF reported in the literature, ranging from 11.2% to 42%, depending on the definition used, and the population studied [9,11,21,22,23].

Secondly, we confirmed the greater responsiveness and reliability of KeGFR in the immediate postoperative period. KeGFR has been specifically designed to estimate GFR when serum creatinine levels are not stable, for example during acute kidney injury [16]. Other biomarkers such as Interleukin-18 [24], liver-type fatty acid-binding protein [25] or Clusterin [13] are intensely studied but none reached clinical use yet [26]. Many studies already pointed out the usefulness of KeGFR in the context of critical illness, where serum creatinine is largely unstable. In a retrospective cohort of patients undergoing cardiac surgery, Seelhamer et al. identified patients who did not have significant changes in serum creatinine levels postoperatively, whereas KeGFR decreased in the same period, and predicted the development of acute kidney injury over the next days [27]. Doyle et al. further demonstrated that KeGFR at the time of admission was a better predictor of acute kidney injury and renal replacement therapy need than classical MDRD estimation [17]. KeGFR was also shown to be a predictor of successful renal replacement therapy discontinuation [28]. To our knowledge, only one retrospective monocentric study investigated the predictive value of KeGFR for delayed graft function in kidney allograft recipients [20]. In this study, 22 transplanted patients out of 56 experienced delayed graft function. The authors showed that adding KeGFR to a validated prediction score for delayed graft function significantly improved diagnostic performance, whereas serum creatine did not. Moreover, prediction discrimination of KeGFR was better than the MDRD formula [20].

We show here that identification of this high-risk population using KeGFR is possible as early as at POD1, independently of the gender, potentially allowing care optimization of these patients. However, our study has some limitations. First, our analyses were retrospective and limited to the recorded data, missing some variables, such as cold ischemia time, immunosuppressive regimen, HLA mismatch ratio or native kidney disease. Nevertheless, these data were not crucial for our study, and the number of excluded patients because of missing data remains low. Second, as a single-center study, we were not able to validate the predictive value of KeGFR in an independent cohort. However, our other findings were consistent with the literature. Third, as there is not a universally accepted definition of the SGF phenotype, our results are limited to the SGF definition we chose, based on recent literature [11]. We further performed sensitivity analyses using the original definition proposed by Humar [9]. Again, we found POD 1 KeGFR to have a better discrimination ability to predict SGF than CKD-EPI estimation using the alternative definition These two definitions have already been found to be associated with higher risk of kidney graft failure [10,11]. Fourth, KeGFR rely on serum creatinine levels that are influenced by many factors other than glomerular filtration rate [29].

Our study has several strengths. First, we included a larger number of patients than most studies published on KeGFR. Second, to our knowledge, this study is the first describing the discriminative value of KeGFR to predict SGF. Third, since we included renal graft recipients from both living and deceased donors, our results are easily transposable to other populations.

## 5. Conclusions

Altogether, we show that KeGFR may be used from the first day after transplantation to identify patients with SGF with good sensibility and specificity, and much more rapidly than CKD-EPI, which should not be used in this situation. Patients with SGF were further shown to have lower renal graft function at one-year follow up.

## Figures and Tables

**Table 1:** Baseline characteristics.

	No SGF (*n* = 250)	SGF (*n* = 76)	Total (*n* = 326)	*p*-Value
Age, median (Q1, Q3)	51.50 (42.00, 62.00)	58.00 (44.75, 68.00)	53.00 (43.00, 63.00)	0.009
Male sex	151 (60.4%)	48 (63.2%)	199 (61.0%)	0.692
Pre-emptive transplantation	37 (14.8%)	4 (5.3%)	41 (12.6%)	0.030
ABO incompatible	21 (7.9%)	3 (3.8%)	24 (6.9%)	0.223
Living donor	119 (47.6%)	12 (15.8%)	131 (40.2%)	<0.001
Donation after cardiac death	3 (1.2%)	8 (10.5%)	11 (3.4%)	<0.001
Donor age, median (Q1, Q3)	52.00 (43.00, 61.00)	57.00 (45.50, 68.50)	53.00 (43.00, 62.50)	0.017
Dialysis	2 (0.8%)	5 (6.6%)	7 (9.6%)	0.003
3 month eGFR, median (Q1, Q3)	56.12 (47.54, 67.66)	41.75 (32.04, 62.65)	54.02 (43.89, 67.32)	<0.001
6 month eGFR, median (Q1, Q3)	54.23 (45.47, 68.59)	46.04 (30.99, 59.97)	53.22 (42.46, 67.69)	<0.001
1 year eGFR, median (Q1, Q3)	56.93 (45.30, 68.57)	46.92 (37.99, 59.90)	53.58 (44.35, 67.73)	0.003
